# Are Viscoelastometric Assays of Old Generation Ready for Disposal? Comment on Volod et al. Viscoelastic Hemostatic Assays: A Primer on Legacy and New Generation Devices. *J. Clin. Med.* 2022, *11*, 860

**DOI:** 10.3390/jcm12020477

**Published:** 2023-01-06

**Authors:** Marion Bareille, Thomas Lecompte, François Mullier, Stéphanie Roullet

**Affiliations:** 1 CHU UCL Namur, Namur Thrombosis and Hemostasis Center (NTHC), Université Catholique de Louvain, 5530 Yvoir, Belgium; 2 Pharmacy Department, University of Namur, 5000 Namur, Belgium; 3 Université de Lorraine, 54000 Nancy, France; 4 Laboratory for Hemostasis, Inflammation & Thrombosis (HITh), Unité Mixte de Recherche (UMR)-1176, Institut National de la Santé et de la Recherche Médicale (Inserm), Université Paris-Saclay, 94270 Le Kremlin-Bicêtre, France

**Keywords:** viscoelastic testing, viscoelastometry, trauma, postpartum hemorrhage, cardiac surgery, liver transplantation, COVID-19, coagulopathy, rotational thromboelastometry, thromboelastography, fibrinogen

## Abstract

With the advent of new viscoelastometric hemostatic assay (VHA) devices, with ready-to-use cartridge reagents allowing for their use by people without special laboratory skills, the appreciation of the actual clinical value of VHAs in settings such as severe trauma, post-partum hemorrhage, cardiac surgery and liver transplantation still needs to be fully validated. While two of the newest versions remain based on a ‘cup and pin’ system (ROTEM^®^ sigma, ClotPro^®^), two other new devices (TEG^®^ 6s, Quantra^®^) rely on very different technologies: clotting blood is no longer in contact with the probe and challenged by oscillation of one of the components but explored with ultrasound exposure. A systematic literature search (including Sonoclot^®^) retrieved 20 observational studies (19 prospective). Most studies pointed to imperfect agreements, highlighting the non-interchangeability of devices. Only a few studies, often with a limited number of patients enrolled, used a clinical outcome. No study compared VHA results with conventional laboratory assays obtained through a rapid tests panel. Clinical evidence of the utility of the new VHAs largely remains to be proven through randomized clinical trials, with clinically relevant outcomes, and compared to rapid panel hemostasis testing. The availability of new, improved VHA devices provides an impetus and an opportunity to do so.

We read with much interest the review by Volod et al. on what they named viscoelastic hemostatic assays (VHAs)—the measurement of changes in mechanical (elastic) properties of a growing and evolving clot of whole blood. The authors are to be commended for the overview of the devices currently available in clinical practice [1]. We would like, however, to raise some concerns about their actual documented clinical usefulness and as a result express a less enthusiastic view.

The authors claim that VHAs have become essential in some surgical settings such as severe trauma, cardiac surgery and liver transplantation. Some others have advocated for the use of VHAs in the diagnosis [2] and the perioperative management of patients with inherited fibrinogen disorders [3]. Even though we do not deny the educational value of VHAs and their popularity, we would like to dampen the appreciation of their actual clinical value. Cardiac surgery is, indeed, one of the clinical settings where the contribution of VHAs has been the most studied and has shown a potential benefit if integrated in a transfusion algorithm, with a decrease in mortality when a VHA-guided algorithm is preferred to a lab-guided algorithm [4,5,6,7]. In severe trauma, however, only one randomized study found a positive effect on mortality of the VHA-guided transfusion algorithm [8]. Finally, in other settings such as liver transplantation or post-partum hemorrhage, the most important is to use a transfusion algorithm and to not delay antifibrinolytic administration, keeping in mind that VHAs lack sensitivity to hyperfibrinolysis [9,10,11,12].

As stated by the authors, ROTEM^®^ sigma and ClotPro^®^ remain based on a ‘cup and pin’ system, whereas two other new devices are based on very different technologies: the TEG^®^ 6s Hemostasis Analyzer (Haemonetics Corporation, Boston, MA, USA) and the Quantra^®^ device (HemoSonics, LLC, Charlottesville, VA, USA); clotting blood is no longer in contact with the probe and challenged by oscillation of one of the components but explored with ultrasound exposure. Although available for a while, we wish also to consider the Sonoclot^®^ analyzer (Sienco, Morrison, CO, USA), since it too does not rely on the ‘cup and pin’ system: it monitors along clotting the changes in impedance to the movement of a probe inserted in the blood sample and vibrating at an ultrasonic frequency [13]. The critical question as to whether the results of clinical studies conducted with an old generation of VHAs based on a ‘cup and pin’ system can be extrapolated to operating rooms or intensive care units where another device of substantially different technology is implemented is unfortunately left unaddressed by the authors. It is of interest too to look at the comparative performances of the two older versions of VHAs based on a ‘cup and pin’ system: ROTEM^®^ sigma and ClotPro^®^.

We thus performed a systematic literature search in PubMed and Scopus databases, using the following keywords: “ROTEM^®^ sigma OR TEG^®^ 6s OR Quantra^®^ OR Sonoclot^®^ OR ClotPro^®^” AND “trauma OR postpartum hemorrhage OR cardiac surgery OR liver transplantation”, to assess the level of evidence currently available for the use of those devices in operating rooms and/or intensive care units. The last search was conducted on 12 August 2022 and included all studies published whether in English, French or Italian. Among the 20 retrieved studies, all observational, 19 were prospective, and most of them were conducted in the field of cardiac surgery (N = 13); three evaluated VHAs in the field of trauma, two studied VHAs during post-partum hemorrhage, and two during liver transplantation (Table). The comparator was variablean older VHA (of the same company (ROTEM devices, TEG devices) or not) and/or a new one as well, with sometimes standard laboratory results and/or clinical endpoints, among which was Clauss fibrinogen. Of note, we found that most studies with Sonoclot^®^ were performed in cardiac surgery (four out of the five retrieved studies); ROTEM^®^ sigma was the only one studied during post-partum hemorrhage (in total, six studies with ROTEM^®^ sigma); TEG^®^ and Quantra^®^ devices were studied five times (across all settings but post-partum hemorrhage and only trauma and cardiac surgery, respectively), whereas ClotPro^®^ was just once, and with the tPA test only (liver transplantation). The way the comparison was analyzed was variable (see Table 1); of note, only parameters dealing with the same aspect of clotting blood in response to a similar initiation (tissue factor or contact phase activator), or with fibrinogen levels, should be compared.

Overall, the studies often pointed to imperfect agreements, often only moderate, particularly when results were outside the normal ranges; thus, the devices were often deemed not interchangeable. Each device and/or clinical setting needs their own cut-off values and algorithms. It would have been more relevant to analyze the number of cases with such a disagreement that the clinical decision would have been affected based on algorithms with cut-off values. A better agreement is expected between devices of the same brand, or between devices relying on the old ‘cup-and-pin’ system. It should be pointed out that the conclusions of the two studies that compared the two ROTEM^®^ devices delta and sigma—one in trauma [15], one in PPH [16]—out of the six studies with ROTEM^®^ sigma, substantially differed: there was agreement that ROTEM^®^-based algorithms may be transposed from a trauma center to another for the former [15], whereas for the latter, ROTEM^®^-based algorithms should be based on device-specific reference values [16].

Of note, only a few studies [19,21,25,27], often with a limited number of patients enrolled, used a clinical outcome in terms of bleeding, transfusion needs, or survival. Randomized controlled trials are still needed to confirm the clinical utility of the new devices. Moreover, none of these studies compared VHA results with conventional laboratory assays obtained through a rapid tests panel. Indeed, clinically relevant and reliable results suitable for acute patient care can be obtained in 20 min through a specific and dedicated laboratory process [34,35]. Owing to the importance ascribed to rapid fibrinogen assessment, the most important point to be checked before implementing a new device is the comparison with values obtained either in the laboratory (Clauss method) and/or the hitherto used VHA device, depending on the local environment. In this regard, such testing could also be of interest to monitor fibrinogen supplementation in patients with inherited fibrinogen disorders.

Another important aspect regards whether internal quality control is provided.

A noticeable asset of TEG^®^ 6s and Quantra^®^ devices, as well as of ClotPro^®^ (Haemonetics Corporation, Boston, MA, USA) and ROTEM^®^ sigma ones, is that they are fully automated, thus making their use very convenient by eliminating pipetting stages. On the other hand, research protocols to investigate what is at stake during normal and disordered changes in viscoelastic properties of a clotting blood sample are rendered more difficult, or even impossible to conduct, since it is no longer possible to vary the experimental conditions for clotting.

As discussed by the authors, VHAs present some limitations. Besides the lack of consideration of the plasma inhibitors of coagulation and of the endothelial components of the hemostatic system (collagen, thrombomodulin, and so forth) [36], one must keep in mind that VHAs lack sensitivity towards hyperfibrinolysis [10,11,12], which is a major concern in trauma, liver transplantation, cardiac surgery and post-partum hemorrhage. Moreover, the ability of those devices to fully assess platelet function is questionable (in particular, the pre-operative estimation of the residual effect of anti-platelet therapy), even with the sophisticated TEG^®^ Platelet Mapping approach [37]. 

The authors devote a large part of the discussion to the COVID-19-related so-called ‘coagulopathy’ (it should in fact be named disordered hemostasis, since all its components are affected). Indeed, severe COVID-19 patients can exhibit both thrombotic and hemorrhagic complications. If VHAs contributed to the study of this ‘coagulopathy’ to some extent (keeping in mind that VHA assays are very sensitive to high fibrinogen plasma levels), that they helped manage is in our opinion an overstatement [38]. Overall, to the best of our knowledge, no firm evidence currently exists about the diagnostic and treatment of what can be considered hypercoagulability with any VHA.

Lastly, the assessment of anticoagulant drugs is mostly restricted to the gross appreciation of residual heparinization post cardiopulmonary bypass cardiac surgery, apart from the dedicated cartridges for direct oral anticoagulants supplied with the ClotPro^®^ device [39].

To conclude, clinical evidence of the utility of VHAs largely remains to be proven through randomized clinical trials, with clinically relevant outcomes, and compared to rapid panel hemostasis testing. The availability of new, improved VHA devices provides an impetus and an opportunity to do so, at last.

## Figures and Tables

**Table 1:** Systematic short summary of retrieved studies comparing devices relying or not on the ‘cup and pin’ system, and new- and old-generation devices relying on the ‘cup and pin’ system.

Article	Setting	Study Objective	Design	N Patients	N blood Samplings	Comparison	Comparison Methods	Study Limitations	Conclusion of the Study
Ziegler *Eur J Anaesthesiol* 2019 [14]	Trauma (level one trauma center)	To evaluate whether TEG^®^ 6s and ROTEM^®^ sigma deliver comparable results	Prospective observational	67	105 (1 up to 3 per patient)	TEG 6s^®^ vs. ROTEM^®^ sigma vs. Clauss fibrinogen	Correlations between TEG^®^ 6s and ROTEM^®^ Sigma measurements calculated using Spearman rank correlation. Differences between categorical variables analyzed using Fisher exact test (χ^2^), and differences between continuous variables tested using the paired *t*-test or the Wilcoxon matched-pairs signed rank test as appropriate.	Few patients with clinically significant thrombocytopenia and low fibrinogen levels; Comparisons not separated according to the sampling time (ER, OR, ICU)	Similar values for maximum clot strength between the two devices but significant differences for the other parameters; Numbers of patients with measurements outside the normal ranges differed significantly
Michelson *Trauma Surg Acute Care Open* 2020 [15]	Trauma (level one trauma centers)	To assess the ability of the Quantra^®^ QStat system to detect ‘coagulopathy’ (including hyperfibrinolysis) with a comparison to ROTEM^®^	Multicenter prospective observational	56	1 up to 2 or 3 per patient (unclear)	Quantra^®^ QStat system vs. ROTEM^®^ delta	Correlations between Quantra^®^ QStat system and ROTEM^®^ delta measurements assessed using Pearson coefficient of correlation. A simple linear regression model used to evaluate the linear relationship between devices measurements. Clinical concordance analysis performed using a 2 × 2 confusion matrix to determine the agreement between the Quantra^®^ and ROTEM^®^ clot lysis parameters.	Few patients with hyperfibrinolysis; Hyperfibrinolysis diagnosed upon a ROTEM^®^ EXTEM ML > 15% (no comparison with a fibrinolysis assay); Analyses not separated according to the sampling time (ER admission, after the administration of blood products or antifibrinolytic drugs)	Strong correlation (Pearson coefficient of correlation: 0.60 to 0.79) between Quantra^®^ QStat and ROTEM^®^ delta parameters; Quantra^®^ QStat system could detect ‘coagulopathies’ associated with critical bleeding in trauma patients
Bouzat *Eur J Trauma Emerg Surg* 2021 [16]	Trauma (level one trauma centers)	To compare the diagnostic performances of ROTEM^®^ delta and ROTEM^®^ sigma for the diagnostic of post-traumatic ‘coagulopathy’	Retrospective analysis of two registries	74 (first center) + 75 (second center)	1 per patient	ROTEM^®^ delta vs. ROTEM^®^ sigma vs. standard laboratory results	AUC-ROC calculated for ROTEM^®^ delta and sigma devices to detect patients with coagulopathy, maximization of the Youden index then used to determine the best threshold and eventually, the AUROCs for the two devices compared with the De Long test	No concomitant measurement of VET parameters with the sigma and the delta ROTEM^®^ devices; Retrospective study	ROTEM^®^-based algorithms may be transposed from a trauma center to another one independently of the ROTEM device in use
Gillissen *Scand J Clin Lab Invest* 2019 [17]	PPH	To compare ROTEM^®^ delta and ROTEM^®^ sigma measurements	Prospective observational	23	26 (1 up to 3 per patient)	ROTEM^®^ delta vs. ROTEM^®^ sigma vs. Clauss fibrinogen	Correlations between ROTEM^®^ delta and ROTEM^®^ sigma measurements assessed using Spearman rank correlation, as well as correlations between FIBTEM values of both ROTEM^®^ devices and Clauss fibrinogen. Statistical significance of the differences between the results from the two devices tested with the Wilcoxon signed rank test.	Limited number of patients enrolled; Few patients with clinically significant low fibrinogen levels	Wide variation between ROTEM^®^ FIBTEM assays performed with both devices, especially in A5 and A10 measurements: ROTEM^®^-based algorithms should be based on device-specific reference values
Bell *Int J Obstet Anesth* 2022 [18]	PPH	To determine the diagnostic performances of ROTEM^®^ sigma for the diagnostic of ‘coagulopathy’ and to assess the impact of a ROTEM^®^-based algorithm on transfusion of blood products	Prospective observational study	521	≥1 per patient	ROTEM^®^ sigma vs. standard laboratory results	Correlations between ROTEM^®^ sigma measurements and standard laboratory results assessed using Pearson coefficient of correlation AUC-ROC, sensitivity, specificity, PPV and NPV calculated for ROTEM^®^ sigma device to detect patients with coagulopathy	Few patients with clinically significant thrombocytopenia and coagulation factor deficiency; Administration of tranexamic acid prior to blood sampling	Reliable detection of Clauss fibrinogen levels ≤ 2g/L with ROTEM^®^ sigma
Yamada *J Anesth* 2007 [19]	Cardiac surgery, elective, with CPB	To assess the usefulness of Sonoclot^®^ in predicting postoperative hemorrhage	Prospective observational study	41	2 per patient (after heparin administration and before protamine administration)	Sonoclot^®^ vs. excessive bleeding defined as chest tube drainage > than 2 mL·kg^−1^·h^−1^ in 1h during the first 4h after surgery	Statistical analysis performed using two-way repeated analysis of variance, Student’s *t*-test, or the χ^2^ test as appropriate between bleeders and non bleeders	Limited number of patients enrolled; Standard laboratory tests not performed	Sonoclot^®^ analysis performed after CPB could predict abnormal postoperative bleeding
Espinosa *BMC Anesthesiol* 2014 [20]	Cardiac surgery, elective	To evaluate the ability of the TEG^®^, ROTEM^®^ and Sonoclot^®^ instruments to detect changes in hemostasis as assessed with standard laboratory tests	Prospective observational study	35	3 per patient	Sonoclot^®^ vs. ROTEM^®^ delta vs. TEG^®^ 5000 vs. standard laboratory tests	Correlations between Sonoclot^®^, ROTEM^®^ delta and TEG^®^ 5000 measurements vs. standard laboratory tests assessed using Pearson coefficient of correlation	Limited number of patients enrolled	Correlation with standard laboratory results: deemed good (0.60 to 0.79) for TEG^®^ and ROTEM^®^ measurements, but moderate (0.40 to 0.59) for Sonoclot^®^ measurements
Bischof *J Cardiothorac Vasc Anesth* 2015 [21]	Cardiac surgery	To determine if Sonoclot^®^ can predict postoperative bleeding	Prospective observational study	300	2 per patient	Sonoclot^®^ vs. standard laboratory tests vs. chest tube drainage at 4, 8 and 12 h postoperatively	Student *t*-test and χ^2^ test for comparison of continuous and nominal data, as appropriate. To study repeated measurements: analysis of variance with a Bonferroni correction. Modelization (linear regression models, linear mixed effects regression models and random effects models) to identify predictors of bleeding. Model then challenged by calculating the AUC-ROC for patients identified as bleeders	Heterogeneous patient population. Standard laboratory tests not performed	Sonoclot^®^ parameters after heparin reversal were highly predictive for postoperative bleeding
Huffmyer *Anesth Analg* 2016 [22]	Cardiac surgery	To evaluate the correlation between Quantra^®^, ROTEM^®^ delta measurements and standard laboratory tests	Prospective observational study	55	3 per patient	Quantra^®^ QPlus system vs. ROTEM^®^ delta vs. standard laboratory tests	Quantra^®^ QPlus system measurements correlated with corresponding ROTEM^®^ delta and standard laboratory parameters using Pearson coefficient of correlation as well as Spearman rank correlation	Few patients with a ‘coagulopathy’; No excessive bleeding requiring massive transfusion during the study	Significant correlation between Quantra^®^ Qplus and ROTEM^®^ delta parameters as well as with low Clauss fibrinogen levels, and ability to detect residual heparin after cardiac surgery with CPB
Erdoes *PloS One* 2018 [23]	Cardiac surgery	To compare TEG^®^ 6s, ROTEM^®^ delta measurements and standard laboratory tests	Prospective observational study	23	3 per patient	TEG^®^ 6s vs. ROTEM^®^delta vs. standard laboratory tests	TEG^®^ 6s measurements correlated with corresponding ROTEM^®^ delta and standard laboratory parameters using Spearman rank correlation. AUC-ROC calculated to explore the accuracy of MA CFF and MA CKH for fibrinogen deficiency	Limited number of patients enrolled	Only TEG^®^ 6s R (clotting time) of CKH (kaolin with heparinase) could be used during full heparinization for CPB. Before and after CPB both devices showed similar values for maximum clot strength but significant differences for the other parameters. Good diagnostic accuracy for fibrinogen levels lower than 1.5 g/L.
Baryshnikova *J Cardiothorac Vasc Anesth* 2019 [24]	Cardiac surgery	To compare Quantra^®^-derived coagulation parameters with ROTEM^®^ delta, standard laboratory tests and platelet function assessed with MEA (with a focus on platelet contribution to clot stiffness—or strength—PCS, and platelet reactivity)	Prospective observational study	30	2 per patient	Quantra^®^ QPlus system vs. ROTEM^®^ delta vs. standard laboratory tests vs. MEA	Quantra^®^ QPlus system measurements correlated with corresponding ROTEM^®^ delta and standard laboratory parameters with Pearson’s correlation coefficient.	Limited number of patients enrolled; Few patients under platelet P2Y12 inhibitors; No samples during CPB	Strong (r value 0.71–0.90) to very strong (r value 0.91–1.00) correlation between Quantra^®^ Qplus and ROTEM^®^ delta parameters as well as with standard laboratory results. Quantra^®^ Qplus PCS parameter reflects mainly platelet count but also platelet response to ADP (MEA)
Terada *Transfusion* 2019 [25]	Cardiac surgery	To determine the clinical usefulness of TEG^®^ and Sonoclot^®^	Prospective observational study	50	3 per patient	Sonoclot^®^ vs. TEG^®^ 6s vs. standard laboratory tests vs. clinical outcomes *	Sonoclot^®^ measurements correlated with corresponding TEG^®^ 6s and standard laboratory parameters using Spearman rank correlation. Multivariate linear regression analyses performed to evaluate the usefulness of TEG^®^ 6s and Sonoclot^®^ measurements in predicting perioperative total blood loss, postoperative drain bleeding volume and the unit number of platelet transfusions.	Limited number of patients enrolled; Heterogeneous patient population; Few patients with ‘coagulopathy’	Sonoclot^®^ could be useful to predict the risks of postoperative bleeding and platelet transfusion
Wong *Anaesth Intensive Care* 2020 [26]	Cardiac surgery	To evaluate the interchangeability between TEG^®^ 5000 non-citrated results and TEG^®^ 6s citrated results	Prospective observational study	99	2 or 3 per patient	TEG^®^ 5000 non-citrated vs. TEG^®^ 6s citrated	Comparison between TEG^®^ 5000 non-citrated vs. TEG^®^ 6s paired test parameters using Bland–Altman plots. Lin’s concordance coefficient to compare agreement between both devices for measuring the same variable. Clinical concordance analysis performed using McNemar’s test (paired χ^2^) to determine the agreement between both devices.	Large number of tests with TEG^®^ 6s interrupted prior to completion (to allow for further testing); Limited number of functional fibrinogen estimates performed with TEG^®^ 5000 (as non-heparinase functional fibrinogen testing is not part of the authors’ current protocol); Comparisons not separated according to the sampling time (pre or post CPB)	Poor concordance between TEG^®^ 5000 non-citrated and TEG^®^ 6s citrated results particularly in patients with ‘coagulopathy’ resulting in a possible change in treatment recommendation for at least 10% of the enrolled patients: TEG^®^-based algorithms should be based on device-specific reference values
Zghaibe *Anaesthesia* 2020 [27]	Cardiac surgery	To compare Quantra^®^ Qplus system, TEG 5000 measurements and standard laboratory tests and to establish preliminary transfusion thresholds for Quantra^®^ Qplus parameters	Prospective observational study	52	3 per patient	Quantra^®^ QPlus system vs. TEG^®^ 5000 vs. standard laboratory tests	Linear regression equations to determine provisional Quantra^®^-based transfusion thresholds from thresholds of corresponding TEG and laboratory tests currently used (local transfusion protocol). Concordance between Quantra^®^ QPlus parameters and corresponding TEG or laboratory parameters at their equivalent thresholds analyzed by 2 × 2 contingency tables. Association of tested parameters with blood product use assessed by ROC analysis.	Low number of patients with coagulopathy	Quantra^®^ Qplus system could be used during CPB and full heparinization as Quantra^®^ QPlus cartridges contain a heparin inhibitor (whereas heparinase has to be added specifically when using TEG^®^ 5000). Using specific TEG^®^ 5000-derived thresholds Quantra^®^ Qplus system showed a high negative predictive value and a low positive predictive value for transfusion, and could be of interest to detect platelet dysfunction due to antiplatelet therapy
Baulig *BMC Anesthesiol* 2021 [28]	Cardiac surgery	To compare Quantra^®^ QPlus system and ROTEM^®^ sigma measurements	Prospective observational study	38	2 per patient	Quantra^®^ QPlus system vs. ROTEM sigma	Correlations between Quantra^®^ QPlus system and ROTEM^®^ sigma measurements assessed using Spearman rank correlation. Comparison between Quantra^®^ QPlus system and ROTEM^®^ sigma parameters using Bland–Altman plots.	Limited number of patients enrolled	Strong (r = 0.70–0.89) correlation between Quantra^®^ QPlus and ROTEM sigma parameters, but with no direct interchangeability between the two devices: separate cut-off values need to be established for Quantra^®^-based algorithms
DeAnda *J Cardiothorac Vasc Anesth* 2021 [29]	Cardiac surgery	To evaluate the correlation and agreement between Quantra^®^ QPlus system, TEG^®^ 5000 and standard laboratory tests	Prospective observational study	28	3 per patient	Quantra^®^ QPlus system vs. TEG^®^ 5000 vs. standard laboratory tests	Quantra^®^ QPlus system measurements were correlated with corresponding TEG5000 parameters using Pearson’s correlation coefficient. The parameters from the Quantra^®^ compared with TEG^®^ 5000, using weighted Deming regression analysis	Limited number of patients enrolled	Strong (0.60 to 0.79) correlation between Quantra^®^ QPlus and TEG^®^ 5000 parameters, but with no direct interchangeability between the two devices: separate cut-off values need to be established for Quantra^®^-based algorithms
Kammerer *Transfus Med Hemother* 2021 [30]	Cardiac surgery	To evaluate the correlation between ClotPro^®^ tPA test (challenge of clotting blood to added tPA) and TXA plasma levels	Prospective observational study	25	7 per patient	ClotPro^®^ t-PA test vs. standard laboratory tests vs. UHPLC-MS/MS for TXA plasma levels vs. PAI-1 antigen and activity levels and t-PA antigen levels	Correlations between VHA parameters and TXA plasma levels assessed using Spearman’s correlation	TXA dosing was at the upper limit of the dose recommendation (50 mg/kg BW); No comparison with a functional assay for fibrinolysis	Some correlation between TXA plasma levels and ClotPro^®^ t-PA test results, with a marked interindividual variability of TXA effects using the ClotPro^®^ t-PA test related to patients’ renal function: the ClotPro^®^ t-PA test could be of interest for individualized dose adjustments of TXA
Preuss *Anaesthesia* 2022 [31]	Cardiac surgery	To evaluate the diagnostic performance of ROTEM^®^ sigma for the identification of low fibrinogen levels	Prospective observational study	120	1 per patient	ROTEM^®^ sigma vs. Clauss fibrinogen	Sensitivity, specificity, PPV and NPV of FIBTEM A5 ≤ 6 mm and A10 ≤ 8 mm with and without the criteria of a FIBTEM/EXTEM clotting time ratio > 1.0 assessed for the identification of laboratory fibrinogen < 1.5 g.L^−1^	Few patients with low fibrinogen levels of clinical significance	FIBTEM/EXTEM clotting time ratio > 1.0 in addition to standard FIBTEM amplitude criteria (A5 ≤ 6 mm or A10 ≤ 8 mm) might improve ROTEM diagnostic accuracy for identification of low fibrinogen levels
Bindi *Minerva Anestesiol* 2001 [32]	Liver transplantation	To evaluate the reliability of Sonoclot^®^ to monitor hemostasis	Prospective observational study	51 patients	3 per patient	Sonoclot^®^ vs. standard laboratory tests	Correlations between Sonoclot^®^ parameters and standard laboratory tests assessed using Pearson’s correlation Ability to detect dysfibrinogenemia and hyperfibrinolysis conditions assessed using Pearson χ^2^ or Fisher test, as appropriate	D-dimer levels as marker of potential hyperfibrinolysis (no comparison with a functional assay for fibrinolysis)	Low to moderate correlation between Sonoclot^®^ parameters and standard laboratory tests. Sonoclot^®^ could be of interest to identify patients with suspected hyperfibrinolysis
Robson *Anaesth Intensive Care* 2019 [33]	Liver transplantation	To evaluate clinical agreement and correlation between thrombelastographic parameters obtained with TEG^®^ 6s and TEG^®^ 5000 devices	Prospective observational study	10	6 per patient	TEG^®^ 5000 citrated and non-citrated vs. TEG^®^ 6s	Comparison between TEG^®^ 5000 citrated and non-citrated vs. TEG^®^ 6s paired test parameters using Bland–Altman plots. Lin’s concordance coefficient to compare agreement between the three assays for measuring the same variable.	Small monocenter study; Comparisons not separated according to liver transplantation phase	Results are not interchangeable as there is often significant disagreement, particularly with results outside the normal ranges

Studies are ranked by settings and year of publication. Analyses were performed with citrated blood samples, unless otherwise specified. Quantra QStat system differs from Quantra QPlus system by exploring fibrinolysis based on the effect of added tranexamic acid. Clinical outcomes (*) in study ref#17 were postoperative drain bleeding volume, number of platelet transfusions and calculated perioperative total blood loss. Of note, it is debatable to use D-dimer levels as a marker of hyperfibrinolysis (study ref#24). AUC-ROC: area under the curve—receiver operating characteristics; BW: body weight; Clauss fibrinogen: clottable fibrinogen upon exogenous thrombin addition; CPB: cardiopulmonary bypass; ER: emergency room; ICU: intensive care unit; MEA: multiple electrode aggregometry; ML: maximum lysis; NA: not applicable; NPV: negative predictive value; OR: operating room; PCS: platelet contribution to clot stiffness; PPH: postpartum hemorrhage; PPV: positive predictive value; tPA: tissue plasminogen activator; TXA: tranexamic acid.
